# The Nuclear and Mitochondrial Genomes of the Facultatively Eusocial Orchid Bee *Euglossa dilemma*

**DOI:** 10.1534/g3.117.043687

**Published:** 2017-07-12

**Authors:** Philipp Brand, Nicholas Saleh, Hailin Pan, Cai Li, Karen M. Kapheim, Santiago R. Ramírez

**Affiliations:** * Department for Evolution and Ecology, Center for Population Biology, and University of California, Davis, California 95616; † Graduate Group in Population Biology, University of California, Davis, California 95616; ‡ China National Genebank, Beijing Genomics Institute, Shenzhen 518083, China; § Department of Biology, Utah State University, Logan, Utah 84322

**Keywords:** whole-genome assembly, corbiculate bee, orchid bee, eusocial, mitochondrial genome, Genome Report

## Abstract

Bees provide indispensable pollination services to both agricultural crops and wild plant populations, and several species of bees have become important models for the study of learning and memory, plant–insect interactions, and social behavior. Orchid bees (Apidae: Euglossini) are especially important to the fields of pollination ecology, evolution, and species conservation. Here we report the nuclear and mitochondrial genome sequences of the orchid bee *Euglossa dilemma* Bembé & Eltz. *E. dilemma* was selected because it is widely distributed, highly abundant, and it was recently naturalized in the southeastern United States. We provide a high-quality assembly of the 3.3 Gb genome, and an official gene set of 15,904 gene annotations. We find high conservation of gene synteny with the honey bee throughout 80 MY of divergence time. This genomic resource represents the first draft genome of the orchid bee genus *Euglossa*, and the first draft orchid bee mitochondrial genome, thus representing a valuable resource to the research community.

Bees (Apoidea) are important models for the study of learning and memory (Menzel and Muller 1996), plant–insect interactions (Doetterl and Vereecken 2010), and the evolution of social behavior (Nowak *et al.* 2010; Woodard *et al.* 2011; Kapheim *et al.* 2015). Among the >20,000 bee species worldwide, lineages have evolved varied degrees of specialization on floral resources such as pollen, resins, and oils (Wcislo and Cane 1996; Michener 2007; Litman *et al.* 2011). These relationships are wide-ranging and have substantial impact on the health and function of natural and agricultural systems (Klein *et al.* 2007). Furthermore, several transitions from an ancestral solitary to a derived eusocial behavior have occurred within bees (Danforth 2002; Cardinal and Danforth 2011; Branstetter *et al.* 2017). Thus, bees provide unique opportunities to investigate the genetic underpinnings of multiple major ecological and evolutionary transitions. The repeated evolution of different behavioral phenotypes in bees, including foraging and social behavior, provides a natural experiment that allows for the determination of general as well as species-specific molecular genomic changes underlying phenotypic transitions. In order to capitalize on this potential, whole-genome sequences of a divergent array of bee species with different life histories are needed (Kapheim *et al.* 2015).

Orchid bees (Apidae; Euglossini) are among the most important pollinators of angiosperms in the neotropical region (Ramírez *et al.* 2002). While female orchid bees collect nectar, pollen, and resin for nest construction and brood-cell provisioning, male bees collect perfume compounds from floral and nonfloral sources (Vogel 1966; Whitten *et al.* 1993; Eltz *et al.* 1999; Roubik and Hanson 2004). These volatile compounds are used to concoct a species-specific perfume blend that is subsequently used during courtship display, presumably to attract conspecific females. This unique male scent-collecting behavior has recently been examined in a broad array of molecular ecological and evolutionary studies, focusing on phenotypic evolution, chemical communication, plant–insect mutualisms, and speciation (Eltz *et al.* 2008, 2011; Ramírez *et al.* 2011; Brand *et al.* 2015; Weber *et al.* 2016).

While most of the ∼220 species of orchid bees appear to be solitary, several species have transitioned to living in coordinated social groups (Garófalo 1985; Pech *et al.* 2008; Augusto and Garófalo 2009). Female *Euglossa dilemma* individuals, for example, can either form solitary nests and provision their own brood cells, or live in small groups where daughters remain in their natal nest and help their mother rear her offspring, instead of dispersing to found their own nest. The social *E. dilemma* nests (similar to the closely related *E. viridissima*; Pech *et al.* 2008) exhibit true division of labor, with subordinate daughters foraging for resources and the reproductively dominant mothers remaining in the nest and laying eggs (N. Saleh and S.R. Ramírez, personal observation). This facultative eusocial behavior represents an early stage in social evolution and makes *E. dilemma* well suited for studying the genetic mechanisms underlying the transition from solitary to eusocial behavior. While other facultative eusocial species evolved throughout the bee lineage, orchid bees have a unique taxonomic position (Cardinal and Danforth 2011). Orchid bees are part of the corbiculate bees, together with the honey bees, bumble bees, and stingless bees, three obligately eusocial bee lineages (Figure 1A). As the sister group to all other corbiculate bee lineages (Romiguier *et al.* 2016; Branstetter *et al.* 2017; Peters *et al.* 2017), orchid bees may provide key insights into the early stages of eusociality and the possible evolutionary trajectories that led to the obligate eusocial behavior exhibited by honey bees.

**Figure 1:**
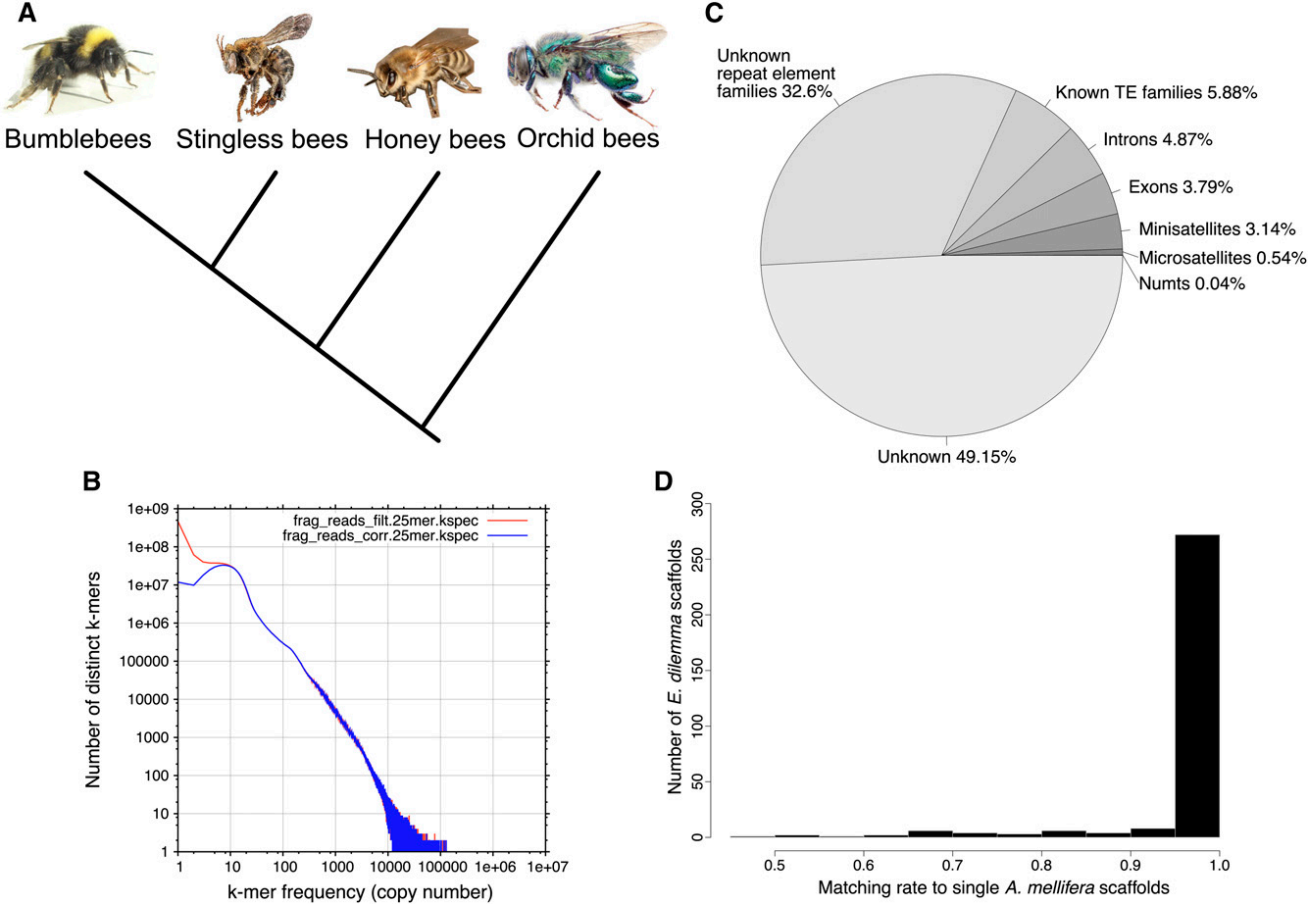
Genomic features. (A) Phylogeny of the four corbiculate bee tribes with orchid bees as sistergroup to honey bees, stingless bees, and bumblebees (Romiguer *et al.* 2016). (B) K-mer distribution spectrum (*k* = 25) of genomic sequence reads. The positively skewed spectrum reveals a high abundance of a few k-mers, leading to an estimate of 87.7% repetitiveness of the *E. dilemma* genome. Red shows the k-mer spectrum before, and blue after error correction. (C) Genomic element density including genic and nongenic features as a fraction of the overall genome assembly length excluding stretches of N. 49.15% of the assembly could not be annotated with the selected methods. (D) Synteny between the *E. dilemma* and the honey bee (*Apis mellifera*) genome. In an analysis including *E. dilemma* scaffolds of ≥100 kb length, 83% showed ≥95% synteny to a single honey bee scaffold. Photographs in (A) are reproduced from Wikimedia under the CC BY-SA 3.0 license.

Here we present the draft genome of the orchid bee species *E. dilemma*. Using a combined paired-end and mate-pair library sequencing approach, we assembled 18% of the predicted 3.3 Gb genome, and annotated a high-quality gene set including 15,904 genes. In addition, we reconstructed three quarters of the mitochondrial genome with the help of transcriptome data, representing the first orchid bee mitogenome. These genomic resources will facilitate the genetic study of outstanding ecological and evolutionary questions, such as the evolution of resource preferences and the evolution of eusociality. Moreover, it provides an important genomic resource for a group of crucial neotropical pollinators, which are of specific concern for conservation biologists (Zimmermann *et al.* 2011; Suni and Brosi 2012; Soro *et al.* 2016; Suni 2016).

## Materials and Methods

### Genome sequencing and assembly

#### Nuclear genome:

Sequencing of the *E. dilemma* genome was based on six haploid male individuals collected at Fern Forest Nature Center in Broward County, FL (26°13’46.3’’N, 80°11’08.9’’W) in February 2011. This population was chosen based on its low nucleotide diversity resulting from a bottleneck during a single introduction to Southern Florida ∼15 yr ago (Skov and Wiley 2005; Pemberton and Wheeler 2006; Zimmermann *et al.* 2011). DNA was extracted from each bee independently and used for the construction of four paired-end (two 170 and 500 bp libraries, respectively) and four mate-pair (two 2 and 5 kb libraries, respectively) sequencing libraries. Next, the paired-end libraries were sequenced in 90 cycles and the mate-pair libraries in 49 cycles on an Illumina HiSeq2000. The resulting sequence data were run through fastuniq v1.1 (Xu *et al.* 2012) to remove polymerase chain reaction (PCR) duplicates and quality trimmed using trim_galore v0.3.7 (Babraham Bioinformatics). Subsequently, reads were used for *de novo* assembly with ALLPATHS-LG v51750 (Gnerre *et al.* 2011) and Soap-denovo2 (Luo *et al.* 2012) with varying settings. Gaps within scaffolds were closed using GapCloser v1.12 (Luo *et al.* 2012) for each assembly. ALLPATHS-LG with default settings resulted in the highest-quality assembly, based on assessments of annotation completeness (see below). This assembly (*E. dilemma* genome assembly v1.0) was used for all subsequent analyses. All other assemblies were excluded from analysis, but are available upon request.

The preprocessed reads were used for k-mer based genome size estimates. We used ALLPATHS-LG to produce and analyze the k-mer frequency spectrum (*k* = 25). Genome size was estimated on the basis of the consecutive length of all reads divided by the overall sequencing depth as (*N* × (*L* − *K* + 1) − *B*)/*D* = *G*, where *N* is the total number of reads, *L* is the single-read length, *K* is the k-mer length, *B* is the total count of low-frequency (frequency ≤3) k-mers that are most likely due to sequencing errors, *D* is the k-mer depth estimated from the k-mer frequency spectrum, and *G* is the genome size. In addition, we used the ALLPATHS-LG k-mer frequency spectrum to predict the repetitive fraction of the genome.

The quality of the genome assembly was assessed using standard N statistics and assembly completeness as measured by the CEGMA v2.5 (Parra *et al.* 2007) and BUSCO v1.1 (Simão *et al.* 2015) pipelines. CEGMA was run in default mode, whereas BUSCO was run with the arthropoda_odb9 OrthoDB database (Zdobnov *et al.* 2017) in genome mode.

We estimated the mean per-base genome coverage on the basis of the preprocessed reads and the estimated genome size as $\sum_{i=1}^{4}\left(R_{i}*L_{i}\right)/G=C,$ where *R* is the number of reads and *L* the mean read length of sequence library *i*, *G* is the estimated genome size, and *C* the resulting per-base coverage.

#### Mitogenome:

Initial attempts to reconstruct the mitochondrial genome from our whole-genome shotgun sequencing reads including read subsampling and exclusion of rare variants were only partially successful, due to high sequence variability of sequencing reads with similarity to mitochondrial loci (data not shown). In addition, we have observed that the amplification of mitochondrial DNA in standard PCR leads to a high level of polymorphic sites in *E. dilemma* and other orchid bees (P. Brand and S.R. Ramírez personal observation). Together, this suggests the presence of nuclear copies of the mitochondrial genome (NUMTs) that interfere with the assembly process and PCR amplification. Mitochondrial genes are expressed in almost every tissue in eukaryotes. We used this feature to reconstruct the mitochondrial genome as far as possible from RNA-Seq data. Therefore, we used available *E. dilemma* transcriptome assemblies in order to reconstruct the mitochondrial genome from cDNA (Brand *et al.* 2015). In order to find expressed mitochondrial genes represented in the transcriptome assembly of Brand *et al.* (2015), we used blastx with the honey bee mitochondrial genome as query (Crozier and Crozier 1993) and an *E*-value cutoff of 10E−12 (Altschul *et al.* 1990; Camacho *et al.* 2009). The contigs and scaffolds that were detected with this approach were annotated following Dietz *et al.* (2016). Briefly, we performed tblastn and tblastx searches with protein-coding genes and rRNA genes of the honey bee mitochondrial genome, respectively. All hits were used for manual gene annotation using Geneious v8.0.5 (Biomatters Ltd. 2012). Since the recovered mitochondrial mRNA scaffolds contained >1 gene, we searched and annotated intergenic tRNAs using ARWEN 1.2.3 (Laslett and Canbäck 2008) and tRNAscan-SE 1.21 (Lowe and Eddy 1997).

### Genome annotation

#### Gene annotation:

Genes were annotated based on sequence homology and *de novo* gene predictions. The homology approach was based on the recently updated high-quality official gene set of the honey bee (OGS v3.2; Elsik *et al.* 2014). All honey bee original gene set (OGS) proteins were used in initial tblastn searches against all *E. dilemma* scaffolds with an *E*-value cutoff of 10E−4. All honey bee protein sequences with a blast hit to the *E. dilemma* genome assembly covering at least 50% of the protein were selected for homology-based annotation. The resulting set of honey bee proteins was used as input to exonerate v2.42.1 (Slater and Birney 2005) in order to annotate homologous open reading frames (ORFs) through accurate exon–intron boundary prediction for each scaffold. Exonerate was run with default settings and the minimum fraction of the possible optimal similarity per protein query set to 35%. In a second round, genes not annotated under the previous settings were rerun with minimum similarity set to 15%. In the case of overlapping annotations on the same strand (*i.e.*, identical ORF orientation) resulting from honey bee queries with high similarity, we discarded all but one annotation with the best exonerate score (based on completeness and similarity). This approach proved feasible due to the relatedness of *E. dilemma* and the honey bee, as well as the high quality of the well curated honey bee OGS. For *de novo* gene prediction we used Augustus (Stanke *et al.* 2008) and SNAP (Korf 2004) trained on the honey bee, with the *E. dilemma* genome masked for repetitive regions (see below) as input. Only genes predicted by both programs were taken into account. Gene predictions with ≥85% sequence similarity to each other were discarded, to prevent the inclusion of putative unmasked transposable element derived genes in the official gene set. *De novo* predictions were added to the *E. dilemma* OGS if not annotated by the homology-based approach.

#### Repetitive element annotation:

Repetitive elements including tandem repeats, nuclear copies of the mitochondrial genome (NUMTs), and transposable elements (TEs) were annotated using multiple methods.

#### Tandem repeats:

We searched for micro- and mini-satellites (1–6 and 7–1000 bp motif length, respectively) in all scaffolds using Phobos 3.3.12 (Mayer 2010). We performed two independent runs for each class of tandem repeats with Phobos parameter settings following Leese *et al.* (2012) (gap score and mismatch score set to −4 and a minimum repeat score of 12; Leese *et al.* 2012).

#### NUMTs:

We annotated NUMTs using blastn runs with the partial mitochondrial genome (see above) as query and an *E*-value cutoff of 10E−4 as used in NUMT analyses of other insect genomes (Pamilo *et al.* 2007). This approach allowed us to find NUMTs with medium to high similarity to the actual transcriptome-based mitochondrial genome.

#### TEs:

In order to annotate TEs, we first ran RepeatModeler for *de novo* repeat element annotation and classification based on the genome annotation followed by RepeatMasker in order to detect the total fraction of repetitive elements present in the genome (Smit *et al.* 2016). In addition we used the k-mer based *de novo* repeat assembler REPdenovo (Chu *et al.* 2016) to identify highly repetitive genomic elements based on the generated short sequence reads. These two methods independently assess repetitive sequence content in genomic data using different approaches thus allowing for a more robust estimation of repetitive genome content. The incorporation of multiple *de novo* repeat detection pipelines was necessary due to the lack of a bee-specific repeat database. RepeatModeler v1.0.8 was run with default settings using the NCBI blast algorithm (Altschul *et al.* 1990) for repeat element detection. We used the resulting *de novo* repeat element annotations as a database for RepeatMasker v4.0.5 with Crossmatch v0.990329 as the search engine. We ran the analysis in sensitive mode in order to identify the total fraction of repeat elements in the assembly. We excluded low complexity regions and small RNA from the analysis (settings -nolow and -norna). REPdenovo v1.0 was run on all preprocessed genomic short reads in default mode with a minimum repeat frequency of 400× (*i.e.*, the squared mean genome coverage of 20×; see *Results and Discussion* section). We then used Bowtie v2 (Langmead and Salzberg 2012) in the sensitive local alignment mode to map all reads to the resulting contigs. The mapping results were then used to calculate mean per-contig coverage with bamtools (Barnett *et al.* 2011). In order to estimate the fraction of the overall genome that corresponds to these highly abundant contigs, we divided the mean contig coverage by the respective contig length and the mean genome-wide coverage. The sum of the resulting normalized base pair counts divided by the estimated genome size was then used as an estimate of the fraction of the genome containing highly abundant sequences.

### Genome structure

To analyze genome structure, we compared the genome-wide gene synteny of *E. dilemma* and the honey bee. We used the genomic locations of homologous genes (as determined above) of the honey bee and *E. dilemma* scaffolds of at least 100 kb length to build haplotype blocks with a minimum length of 1 kb. Haplotype blocks included the entire gene span as well as intergenic regions whenever two or more adjacent genes were homologous in both species. We discarded gene annotations from downstream analysis that were recovered as homologous to multiple genomic locations in either species. Furthermore, we excluded *E. dilemma* genes that were recovered as homologous to honey bee scaffolds belonging to unknown linkage groups.

### Data availability

The *E. dilemma* genome assembly *Edil_v1.0*, the annotation, and the original gene set *Edil_OGS_v1.0* are available for download via NCBI (Bioproject: PRJNA388474), Beebase (Elsik *et al.* 2016), the i5k NAL workspace (https://i5k.nal.usda.gov/euglossa-dilemma) (i5K Consortium 2013), and the Ramirez Lab website. The raw reads are available via NCBI (Bioproject: PRJNA388474). The published raw transcriptome sequence reads are available at the NCBI Sequence Read Archive (SRA: SRX765918) (Brand *et al.* 2015).

## Results and Discussion

### Whole-genome assembly

The *E. dilemma* genome assembly resulted in 22,698 scaffolds with an N50 scaffold length of 144 Kb and a total length of 588 Mb (Table 1). This represents 18% of the k-mer based estimated genome size of 3.2 Gb. Of all sequence reads, 68% aligned to the genome assembly, of which 56% aligned more than once. Further, the k-mer frequency spectrum based on all sequencing reads was strongly positively skewed indicating the presence of highly repetitive sequences in the read set (Figure 1B). Based on the k-mer frequency spectrum, 87.7% of the genome was estimated to be repetitive. This suggests that the genome of *E. dilemma* consists largely of highly repetitive sequences, explaining the low consecutive assembly length, the high assembly fragmentation, and the high fraction of sequence reads mapping multiple times to the assembly. The mean per-base coverage was estimated to be comparatively low in comparison to previous bee genome assemblies, with 19.7× based on the preprocessed reads and estimated genome size (Kocher *et al.* 2013, 95.65× coverage; Kapheim *et al.* 2015, 120–272.3× coverage). Total genomic GC content was 39.9%, and thus similar to previously sequenced bee genomes ranging between 32.7 and 41.5% (Table 1) (Kocher *et al.* 2013; Elsik *et al.* 2014; Kapheim *et al.* 2015).

**Table 1:** *E*. *dilemma* genome assembly statistics in comparison to previously published bee genomes

Species	N50	N25	Longest Scaffold	Scaffolds	Assembly Length	GC (%)	Predicted Genes	Ref.
*Euglossa dilemma*	143,590	1,417,006	10,108,120	22,698	588,199,720	39.94	15,904	1
*Eufriesea mexicana*	2427	443,231	4,677,300	3,522,543	1,031,837,970	41.38	12,022	2
*Apis mellifera*	997,192	1,922,192	4,736,299	5644	234,070,657	32.70	15,314	3
*Melipona quadrifasciata*	68,085	1,896,322	12,087,087	38,604	507,114,161	38.88	14,257	2
*Bombus impatiens*	1,399,493	2,389,513	5,466,090	5559	249,185,056	37.75	15,896	4
*Lasioglossum albipes*	616,426	1,130,413	3,533,895	41,433	341,616,641	41.50	13,448	5

N50 and N25 indicate the length of the shortest scaffold of those including 50 and 25% of the base pairs in a genome assembly. References (Ref.): 1: this study, 2: Kapheim *et al.* (2015), 3: Elsik *et al.* (2014), 4: Sadd *et al.* (2015), 5: Kocher *et al.* (2012).

Despite the fragmentation of the genome assembly representing <20% of the estimated genome size, CEGMA analysis revealed complete assemblies of 231 out of 248 core eukaryotic genes (93.2% completeness). Similarly, BUSCO analysis revealed that 1007 out of 1066 highly conserved arthropod genes were completely assembled (94.4% completeness). The BUSCO analysis detected the duplication of a fraction of 4.8% of the benchmark single-copy ortholog genes in the genome, which is similar to the fraction of arthropod single-copy orthologs found to be duplicated in the honey bee genome (6.9%, Weinstock *et al.* 2006).

Our gene prediction approach generated a comprehensive official gene set including 15,904 protein-coding genes (Table 1). Of these gene models, 11,139 were derived from homology-based predictions, representing 73% of the 15,314 honey bee genes used for annotation. These annotations are well within or exceeding previous bee genome assemblies and are similar to those reported for the other orchid bee genomes available (Table 1) (Kocher *et al.* 2013; Elsik *et al.* 2014; Kapheim *et al.* 2015; Park *et al.* 2015; Sadd *et al.* 2015).

The CEGMA and BUSCO analysis and the gene annotation results suggest that the gene-coding fraction of the *E. dilemma* genome was properly assembled, despite the large estimated genome size and comparatively low per-base sequencing coverage. However, genetic material obtained from natural populations as in our study can lead to the fragmentation of assemblies due to high nucleotide diversity (Kajitani *et al.* 2014). In addition, high genetic diversity in the underlying genetic material can lead to a high number of false duplicates due to multiple incorporation of divergent genomic regions in genome assemblies (Kelley and Salzberg 2010). Our BUSCO analysis suggests that the assembly did not produce an unusually high fraction of duplicated benchmark single-copy orthologs, indicating a relatively low abundance of false duplicates. The observed fragmentation in our assembly is thus likely to be primarily the result of repetitive genomic elements, and less likely the result of low coverage or high nucleotide diversity in the genetic material used for sequencing.

Overall, our results suggest that our approach was sufficient to produce a high-quality official gene set. The homology-based approach we used resulted in the majority of annotated genes in the official gene set with a known homology to honey bee genes (Supplemental Material, Table S1). This genomic resource will facilitate genome-wide expression studies including gene ontology analyses and comparative gene set analyses among insects.

### Mitochondrial genome assembly

**Figure 2:**
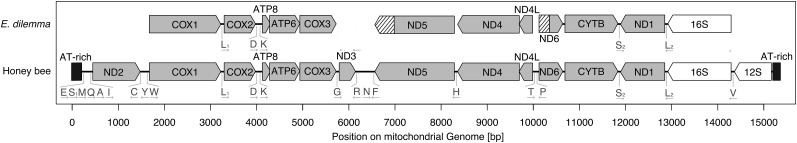
Mitochondrial genome reconstruction. The structure of the honey bee mitochondrial genome and information of the homologous reconstructed parts of the *E. dilemma* mitochondrial genome. Nonreconstructed parts of incompletely reconstructed genes are hatched.

The recently published transcriptome assembly used for the reconstruction of the mitochondrial genome contained four scaffolds between 1222 and 4188 bp long with a total consecutive length of 11,128 bp (Figure 2). This corresponds to ∼75% of the estimated length of the mitochondrial genome, based on other corbiculate bee species (Crozier and Crozier 1993; Cha *et al.* 2007). The *E. dilemma* mitogenome fragments contained 5 out of 22 tRNAs, 11 out of 13 protein-coding genes of which two were only partially recovered, and the 16S rRNA gene. Within scaffolds all genes showed the known hymenopteran gene order and orientation, while the orientation of the five tRNAs detected was identical to those in the honey bee (Crozier and Crozier 1993; Cha *et al.* 2007). Attempts to complete the mitochondrial genome using the nuclear genome assembly yielded no improvement of the assembly (data not shown). Accordingly, the mitochondrial genome is entirely derived from transcriptomic sequences.

The high success in mitochondrial gene reconstruction is likely due to the nature of the analyzed transcriptome data. Short intergenic regions as well as polycistronic mitochondrial mRNA likely lead to the assembly of multiple genes into single scaffolds. The A-T rich region is completely missing as well as the ND2 and 12S rRNA genes flanking the region in insect mitogenomes. This unrecovered region also contains a high number of tRNAs in the honey bee, which could explain the low number of recovered tRNAs in *E. dilemma*. While the partial mitochondrial genome assembly is only 75% complete, it represents the first mitogenome for the group of orchid bees and will thus be a valuable resource for future phylogenetic analyses within the lineage and between more distantly related bee taxa.

### Repetitive elements

#### Tandem repeats:

We detected 76,001 microsatellite loci with a consecutive length of 2,291,067 bp. Mini-satellites with motif lengths from 7 to 1000 bp were less numerous in the genome (67,323 loci), totaling 13,343,515 bp. Accordingly, tandem repeats represent 3.86% of the genome assembly, suggesting that they contribute only a small proportion to the overall genome size (Figure 1C).

#### NUMTs:

We detected fragments with similarity to the draft mitochondrial genome on 129 scaffolds totaling a length of 150,670 bp. The fragments had a mean length of 764.8 bp and a mean similarity of 91.5% to the mitogenome. This suggests that these fragments are not derived from the mitochondrial genome and represent actual NUMTs. A total of 39 scaffolds carried multiple fragments with high similarity to the mitogenome with a concatenated length of up to 6566 bp, suggesting that respective NUMTs might have originated from larger fragments of the mitogenome. In total, only 0.04% of the whole-genome assembly had hits to the mitogenome (Figure 1C). This is likely an underestimate, due to the incompleteness of the reconstructed mitochondrial genome. Nevertheless, NUMTs likely represent only a small fraction of the whole nuclear *E. dilemma* genome. Previous analyses have shown a high density of NUMTs in the honey bee in comparison to other insect genomes totaling ∼0.1% of the overall genome size (Pamilo *et al.* 2007). Accordingly, given the NUMT content detected in *E. dilemma*, it is possible that a comparatively high NUMT density is a common feature of corbiculate bee genomes.

#### TEs:

The RepeatModeler analysis revealed a total of 566 repeat element families in the genome assembly of which 142 (25.1%) belonged to known TE families including 106 DNA transposons and 36 retroelements, while the remaining 424 (74.9%) repeat element families could not be classified into known TE families (Table 2 and Table S2). Using the 566 newly detected repeat element families as the input database for RepeatMasker, we annotated a total of 597,369 elements in the genome assembly of which 74,513 (12.5%) were derived from the 142 classified TE families. The remaining 522,856 (87.5%) elements belonged to the unclassified repeat element families. In total, all annotated repeat elements had a cumulative length of 163,384,833 bp corresponding to 38.48% of the total genome assembly. The majority was derived from unclassified (*i.e.*, unknown) repeat element families corresponding to a total of 32.6% of the genome assembly (Figure 1C). Similarly, the read-based *de novo* repeat analysis in REPdenovo revealed 27,636 contigs derived from k-mers with a minimum coverage of 400× (*i.e.*, the squared mean genome-wide coverage), which includes 831,433,228 bp (26%) of the estimated 3.3 Gb genome.

**Table 2:** Transposable element repeat class analysis

Repeat Element Family	Number Unique Elements	Total Number Elements in Assembly	Cumulative Length (bp)	Percent of Genome Assembly*^a^*
Class I – retrotransposons	36	12,989	8,843,560	2.1
Class II – DNA transposons	106	61,524	16,133,727	3.8
Total classified transposons	142	74,513	24,977,287	5.9
Unclassified	424	522,856	138,407,546	32.6
Total repeat families	566	597,369	163,384,833	38.5

a The percentage was calculated excluding all stretches of *N* in the scaffolds.

The detected high fraction of the genome associated with repetitive element families in *E. dilemma* is not surprising given that large genome sizes are often associated with elevated TE activity and TE content. Similar patterns have been observed in the genomes of diverse lineages, ranging from unicellular eukaryotes to complex multicellular organisms like plants, invertebrates and vertebrates (Kidwell 2002). However, TEs are fast evolving and highly specific to their host lineages, which leads to large underestimates of genomic TE content in previously unstudied lineages (Chalopin *et al.* 2015; Platt *et al.* 2016). This likely explains the large fraction of unclassified repetitive element families that we detected in our genome assembly. Further, the remaining high fraction of unknown genome content (49.15%, Figure 1C) may have resulted from undetected repetitive elements with no similarity to elements from other genomes sequenced previously. The only publicly accessible bee repeat element families are derived from the honey bee, a species with a comparatively small genome (0.23 Gb) and low TE diversity and content (Weinstock *et al.* 2006; Kapheim *et al.* 2015). Our efforts to annotate TEs based on known honey bee elements did not improve the TE annotation (data not shown). Overall, our analysis suggests that a large proportion of the *E. dilemma* genome is repetitive. This result is similar to the results obtained in the orchid bee *Eufriesea mexicana*, which has an estimated repetitive genome content of 31% (Kapheim *et al.* 2015).

### Genome structure

Of the 22,698 *E. dilemma* scaffolds, 580 were at least 100 kb in length and used for synteny analysis with the honey bee genome. A total of 356 of these scaffolds carried at least one gene annotation with known homology to the honey bee, and 329 of these *E. dilemma* scaffolds were homologous to honey bee scaffolds with known linkage group (LG) association (Table S1). Of these scaffolds, 272 (83%) showed ≥95% syntenic homology (Figure 1D). Overall, the detected syntenic linkage blocks cover 222 MB of scaffold length with homology to the honey bee, representing 85% of the 329 filtered scaffolds. This suggests that the genomic architecture is very similar between *E. dilemma* and the honey bee, representing a high level of conservation during the 80 MY since the two lineages diverged. Further, our results support a recent comparative analysis of the honey bee and the bumblebee genomes, which revealed high conservation of genomic synteny (Stolle *et al.* 2011). In comparison, in previous studies gene synteny was found to be less conserved in other insect groups. Extensive local shuffling of gene order beginning on the time scale of 20–40 MY evolutionary distance was described for dipterans, moths, and ants resulting in 60–70% genome-wide synteny after ∼60 MY of divergence time in flies and ants (Clark *et al.* 2007; d’Alençon *et al.* 2010; Obbard *et al.* 2012; Simola *et al.* 2013; Neafsey *et al.* 2015; Nygaard *et al.* 2016). Together, these results support a general pattern of slow evolution of gene synteny in corbiculate bees, independent of the fraction of repetitive genome content.

### Conclusion

The genome assembly of the orchid bee *E. dilemma* that we present here is of high quality, despite its large genome size (estimated to be 3.3 Gb). The 15,904 gene annotations provide a comprehensive set of genes with known homology to the honey bee, facilitating future gene ontology and functional genomic analyses. While we were unable to annotate the mostly repetitive majority of the genome assembly with our approach, the provided sequence reads will be useful for future analyses of repetitive genetic elements in the genome. The nuclear and mitochondrial draft genomes represent a valuable genomic resource for the community of bee geneticists. This genomic resource will likely prove valuable in genetic and functional genomic analyses dealing with the ecology, evolution, and conservation of orchid bees. Furthermore, the genome of the facultatively eusocial *E. dilemma* will be helpful in the study of the evolution of eusociality, due to its taxonomic placement as the sister lineage to the three obligately eusocial corbiculate bee tribes including stingless bees, bumblebees, and honey bees.

## Supplementary Material

Supplemental material is available online at www.g3journal.org/lookup/suppl/doi:10.1534/g3.117.043687/-/DC1.

## References

[bib1] Altschul S. F. Gish W. Miller W. Myers E. W. Lipman D. J., 1990 Basic local alignment search tool. J. Mol. Biol. 215: 403–410. 2231712 10.1016/S0022-2836(05)80360-2

[bib2] Augusto S. C. Garófalo C. A., 2009 Bionomics and sociological aspects of Euglossa fimbriata (Apidae, Euglossini). Genet. Mol. Res. 8: 525–538. 19551641

[bib3] Barnett D. W. Garrison E. K. Quinlan A. R. Strömberg M. P. Marth G. T., 2011 BamTools: a C++ API and toolkit for analyzing and managing BAM files. Bioinformatics 27: 1691–1692. 21493652 10.1093/bioinformatics/btr174 PMC3106182

[bib4] Brand P. Ramírez S. R. Leese F. Quezada-Euan J. J. Tollrian R., 2015 Rapid evolution of chemosensory receptor genes in a pair of sibling species of orchid bees (Apidae: Euglossini). BMC Evol. Biol. 15: 176. 26314297 10.1186/s12862-015-0451-9 PMC4552289

[bib5] Branstetter M. G. Danforth B. N. Pitts J. P. Faircloth B. C. Ward P. S., 2017 Phylogenomic insights into the evolution of stinging wasps and the origins of ants and bees. Curr. Biol. 27: 1019–1025. 28376325 10.1016/j.cub.2017.03.027

[bib6] Camacho C. Coulouris G. Avagyan V. Ma N. Papadopoulos J., 2009 BLAST+: architecture and applications. BMC Bioinformatics 10: 421. 20003500 10.1186/1471-2105-10-421 PMC2803857

[bib7] Cardinal S. Danforth B. N., 2011 The antiquity and evolutionary history of social behavior in bees. PLoS One 6: e21086. 21695157 10.1371/journal.pone.0021086 PMC3113908

[bib8] Cha S. Y. Yoon H. J. Lee E. M. Yoon M. H. Hwang J. S., 2007 The complete nucleotide sequence and gene organization of the mitochondrial genome of the bumblebee, Bombus ignitus (Hymenoptera: Apidae). Gene 392: 206–220. 17321076 10.1016/j.gene.2006.12.031

[bib9] Chalopin D. Naville M. Plard F. Galiana D. Volff J.-N., 2015 Comparative analysis of transposable elements highlights mobilome diversity and evolution in vertebrates. Genome Biol. Evol. 7: 567–580. 25577199 10.1093/gbe/evv005 PMC4350176

[bib10] Chu C. Nielsen R. Wu Y., 2016 REPdenovo: inferring de novo repeat motifs from short sequence reads. PLoS One 11: e0150719. 26977803 10.1371/journal.pone.0150719 PMC4792456

[bib11] Clark A. G. Eisen M. B. Smith D. R. Bergman C. M. Oliver B., 2007 Evolution of genes and genomes on the Drosophila phylogeny. Nature 450: 203–218. 17994087 10.1038/nature06341

[bib12] Crozier R. H. Crozier Y. C., 1993 The mitochondrial genome of the honeybee Apis mellifera: complete sequence and genome organization. Genetics 133: 97–117. 8417993 10.1093/genetics/133.1.97 PMC1205303

[bib13] d’Alençon E. Sezutsu H. Legeai F. Permal E. Bernard-Samain S., 2010 Extensive synteny conservation of holocentric chromosomes in Lepidoptera despite high rates of local genome rearrangements. Proc. Natl. Acad. Sci. USA 107: 7680–7685. 20388903 10.1073/pnas.0910413107 PMC2867904

[bib14] Danforth B. N., 2002 Evolution of sociality in a primitively eusocial lineage of bees. Proc. Natl. Acad. Sci. USA 99: 286–290. 11782550 10.1073/pnas.012387999 PMC117553

[bib15] Dietz L. Brand P. Eschner L. M. Leese F., 2016 The mitochondrial genomes of the caddisflies Sericostoma personatum and Thremma gallicum (Insecta: Trichoptera). Mitochondrial DNA A DNA Mapp. Seq. Anal. 27: 3293–3294. 25714156 10.3109/19401736.2015.1015009

[bib16] Doetterl S. Vereecken N. J., 2010 The chemical ecology and evolution of bee–flower interactions: a review and perspectives. Can. J. Zool. 88: 668–697.

[bib17] Elsik C. G. Worley K. C. Bennett A. K. Beye M. Camara F., 2014 Finding the missing honey bee genes: lessons learned from a genome upgrade. BMC Genomics 15: 86. 24479613 10.1186/1471-2164-15-86 PMC4028053

[bib18] Elsik C. G. Tayal A. Diesh C. M. Unni D. R. Emery M. L., 2016 Hymenoptera genome database: integrating genome annotations in HymenopteraMine. Nucleic Acids Res. 44: D793–D800. 26578564 10.1093/nar/gkv1208 PMC4702858

[bib19] Eltz T. Whitten W. M. Roubik D. W. Linsenmair K. E., 1999 Fragrance collection, storage, and accumulation by individual male orchid bees. J. Chem. Ecol. 25: 157–176.

[bib20] Eltz T. Zimmermann Y. Pfeiffer C. Pech J. R. Twele R., 2008 An olfactory shift is associated with male perfume differentiation and species divergence in orchid bees. Curr. Biol. 18: 1844–1848. 19062287 10.1016/j.cub.2008.10.049

[bib21] Eltz T. Fritzsch F. Pech J. R. Zimmermann Y. Ramirez S. R., 2011 Characterization of the orchid bee Euglossa viridissima (Apidae: Euglossini) and a novel cryptic sibling species, by morphological, chemical, and genetic characters. Zool. J. Linnean Soc. 163: 1064–1076.

[bib22] Garófalo C. A., 1985 Social structure of Euglossa cordata nests (Hymenoptera: Apidae: Euglossini). Entomol. Gen. 11: 77–83.

[bib23] Gnerre S. MacCallum I. Przybylski D. Ribeiro F. J. Burton J. N., 2011 High-quality draft assemblies of mammalian genomes from massively parallel sequence data. Proc. Natl. Acad. Sci. USA 108: 1513–1518. 21187386 10.1073/pnas.1017351108 PMC3029755

[bib24] i5K Consortium, 2013 The i5K initiative: advancing arthropod genomics for knowledge, human health, agriculture, and the environment. J. Hered. 104: 595–600. 23940263 10.1093/jhered/est050 PMC4046820

[bib25] Kajitani R. Toshimoto K. Noguchi H. Toyoda A. Ogura Y., 2014 Efficient de novo assembly of highly heterozygous genomes from whole-genome shotgun short reads. Genome Res. 24: 1384–1395. 24755901 10.1101/gr.170720.113 PMC4120091

[bib26] Kapheim K. M. Pan H. Li C. Salzberg S. L. Puiu D., 2015 Social evolution. Genomic signatures of evolutionary transitions from solitary to group living. Science 348: 1139–1143. 25977371 10.1126/science.aaa4788 PMC5471836

[bib27] Kelley D. R. Salzberg S. L., 2010 Detection and correction of false segmental duplications caused by genome mis-assembly. Genome Biol. 11: R28. 20219098 10.1186/gb-2010-11-3-r28 PMC2864568

[bib28] Kidwell M. G., 2002 Transposable elements and the evolution of genome size in eukaryotes. Genetica 115: 49–63. 12188048 10.1023/a:1016072014259

[bib29] Klein A.-M. Vaissière B. E. Cane J. H. Steffan-Dewenter I. Cunningham S. A., 2007 Importance of pollinators in changing landscapes for world crops. Proc. Biol. Sci. 274: 303–313. 17164193 10.1098/rspb.2006.3721 PMC1702377

[bib30] Kocher S. D. Li C. Yang W. Tan H. Yi S. V., 2013 The draft genome of a socially polymorphic halictid bee, Lasioglossum albipes. Genome Biol. 14: R142. 24359881 10.1186/gb-2013-14-12-r142 PMC4062844

[bib31] Korf I., 2004 Gene finding in novel genomes. BMC Bioinformatics 5: 59. 15144565 10.1186/1471-2105-5-59 PMC421630

[bib32] Langmead B. Salzberg S. L., 2012 Fast gapped-read alignment with Bowtie 2. Nat. Methods 9: 357–359. 22388286 10.1038/nmeth.1923 PMC3322381

[bib33] Laslett D. Canbäck B., 2008 ARWEN: a program to detect tRNA genes in metazoan mitochondrial nucleotide sequences. Bioinformatics 24: 172–175. 18033792 10.1093/bioinformatics/btm573

[bib34] Leese F. Brand P. Rozenberg A. Mayer C. Agrawal S., 2012 Exploring Pandora’s box: potential and pitfalls of low coverage genome surveys for evolutionary biology. PLoS One 7: e49202. 23185309 10.1371/journal.pone.0049202 PMC3504011

[bib35] Litman J. R. Danforth B. N. Eardley C. D. Praz C. J., 2011 Why do leafcutter bees cut leaves? New insights into the early evolution of bees. Proc. Biol. Sci. 278: 3593–3600. 21490010 10.1098/rspb.2011.0365 PMC3189370

[bib36] Lowe T. M. Eddy S. R., 1997 tRNAscan-SE: a program for improved detection of transfer RNA genes in genomic sequence. Nucleic Acids Res. 25: 955–964. 9023104 10.1093/nar/25.5.955 PMC146525

[bib37] Luo, R., B. Liu, Y. Xie, Z. Li, W. Huang *et al.*, 2012 SOAPdenovo2: an empirically improved memory-efficient short-read de novo assembler. GigaScience 1: 18 (erratum: GigaScience 4: 30). 10.1186/2047-217X-1-18 PMC3626529 23587118

[bib38] Mayer, C., 2010 Phobos Version 3.3.12. A Tandem Repeat Search Program. 20 p. Available at: http://www.rub.de/spezzoo/cm/cm_phobos.htm. Accessed: September 30, 2016.

[bib39] Menzel R. Muller U., 1996 Learning and memory in honeybees: from behavior to neural substrates. Annu. Rev. Neurosci. 19: 379–404. 8833448 10.1146/annurev.ne.19.030196.002115

[bib40] Michener C. D., 2007 *The Bees of the World*, Ed. 2. The John Hopkins University Press, Baltimore, MD.

[bib41] Neafsey D. E. Waterhouse R. M. Abai M. R. Aganezov S. S. Alekseyev M. A., 2015 Highly evolvable malaria vectors: the genomes of 16 Anopheles mosquitoes. Science 347: 1258522. 25554792 10.1126/science.1258522 PMC4380271

[bib42] Nowak M. A. Tarnita C. E. Wilson E. O., 2010 The evolution of eusociality. Nature 466: 1057–1062. 20740005 10.1038/nature09205 PMC3279739

[bib43] Nygaard S. Hu H. Li C. Schiøtt M. Chen Z., 2016 Reciprocal genomic evolution in the ant–fungus agricultural symbiosis. Nat. Commun. 7: 12233. 27436133 10.1038/ncomms12233 PMC4961791

[bib44] Obbard D. J. Maclennan J. Kim K.-W. Rambaut A. O’Grady P. M., 2012 Estimating divergence dates and substitution rates in the Drosophila phylogeny. Mol. Biol. Evol. 29: 3459–3473. 22683811 10.1093/molbev/mss150 PMC3472498

[bib45] Pamilo P. Viljakainen L. Vihavainen A., 2007 Exceptionally high density of NUMTs in the honeybee genome. Mol. Biol. Evol. 24: 1340–1346. 17383971 10.1093/molbev/msm055

[bib46] Park D. Jung J. W. Choi B.-S. Jayakodi M. Lee J., 2015 Uncovering the novel characteristics of Asian honey bee, Apis cerana, by whole genome sequencing. BMC Genomics 16: 1. 25553907 10.1186/1471-2164-16-1 PMC4326529

[bib47] Parra G. Bradnam K. Korf I., 2007 CEGMA: a pipeline to accurately annotate core genes in eukaryotic genomes. Bioinformatics 23: 1061–1067. 17332020 10.1093/bioinformatics/btm071

[bib48] Pech, M. E. C., W. de J. May-Itzá, L. A. M. Medina, and J. J. G. Quezada-Euan, 2008 Sociality in Euglossa (Euglossa) viridissima Friese (Hymenoptera, Apidae, Euglossini). Insectes Sociaux 55: 428–433.

[bib49] Pemberton R. W. Wheeler G. S., 2006 Orchid bees don’t need orchids: evidence from the naturalization of an orchid bee in Florida. Ecology 87: 1995–2001. 16937639 10.1890/0012-9658(2006)87[1995:obdnoe]2.0.co;2

[bib50] Peters R. S. Krogmann L. Mayer C. Donath A. Gunkel S., 2017 Evolutionary history of the Hymenoptera. Curr. Biol. 27: 1013–1018. 28343967 10.1016/j.cub.2017.01.027

[bib51] Platt R. N. Blanco-Berdugo L. Ray D. A., 2016 Accurate transposable element annotation is vital when analyzing new genome assemblies. Genome Biol. Evol. 8: 403–410. 26802115 10.1093/gbe/evw009 PMC4779615

[bib52] Ramírez S. Dressler R. L. Ospina M., 2002 Abejas euglosinas (Hymenoptera: Apidae) de la región Neotropical: Listado de especies con notas sobre su biología. Biota Colombiana 3: 7–118.

[bib53] Ramírez S. R. Eltz T. Fujiwara M. K. Gerlach G. Goldman-Huertas B., 2011 Asynchronous diversification in a specialized plant-pollinator mutualism. Science 333: 1742–1746. 21940893 10.1126/science.1209175

[bib54] Romiguier J. Cameron S. A. Woodard S. H. Fischman B. J. Keller L., 2016 Phylogenomics controlling for base compositional bias reveals a single origin of eusociality in corbiculate bees. Mol. Biol. Evol. 33: 670–678. 26576851 10.1093/molbev/msv258

[bib55] Roubik D. W. Hanson P. E., 2004 *Orchid Bees of Tropical America: Biology and Field Guide*. Instituto Nacional de Biodiversidad (INBio), Santo Domingo De Heredia, Costa Rica.

[bib56] Sadd B. M. Barribeau S. M. Bloch G. de Graaf D. C. Dearden P., 2015 The genomes of two key bumblebee species with primitive eusocial organization. Genome Biol. 16: 76. 25908251 10.1186/s13059-015-0623-3 PMC4414376

[bib57] Simão F. A. Waterhouse R. M. Ioannidis P. Kriventseva E. V. Zdobnov E. M., 2015 BUSCO: assessing genome assembly and annotation completeness with single-copy orthologs. Bioinformatics 31: 3210–3212. 26059717 10.1093/bioinformatics/btv351

[bib58] Simola D. F. Wissler L. Donahue G. Waterhouse R. M. Helmkampf M., 2013 Social insect genomes exhibit dramatic evolution in gene composition and regulation while preserving regulatory features linked to sociality. Genome Res. 23: 1235–1247. 23636946 10.1101/gr.155408.113 PMC3730098

[bib59] Skov C. Wiley J., 2005 Establishment of the neotropical orchid bee Euglossa viridissima (Hymenoptera: Apidae) in Florida. Fla. Entomol. 88: 225–227.

[bib60] Slater G. S. C. Birney E., 2005 Automated generation of heuristics for biological sequence comparison. BMC Bioinformatics 6: 31. 15713233 10.1186/1471-2105-6-31 PMC553969

[bib75] Smit A. F. A Hubley R. Green P., 2013-2017 *RepeatMasker Open-4.0.* http://www.repeatmasker.org.

[bib61] Soro A. Quezada-Euan J. J. G. Theodorou P. Moritz R. F. A. Paxton R. J., 2016 The population genetics of two orchid bees suggests high dispersal, low diploid male production and only an effect of island isolation in lowering genetic diversity. Conserv. Genet. 18: 607–619.

[bib62] Stanke M. Diekhans M. Baertsch R. Haussler D., 2008 Using native and syntenically mapped cDNA alignments to improve de novo gene finding. Bioinformatics 24: 637–644. 18218656 10.1093/bioinformatics/btn013

[bib63] Stolle E. Wilfert L. Schmid-Hempel R. Schmid-Hempel P. Kube M., 2011 A second generation genetic map of the bumblebee Bombus terrestris (Linnaeus, 1758) reveals slow genome and chromosome evolution in the Apidae. BMC Genomics 12: 48. 21247459 10.1186/1471-2164-12-48 PMC3034698

[bib64] Suni S. S., 2016 Dispersal of the orchid bee Euglossa imperialis over degraded habitat and intact forest. Conserv. Genet. 18: 621–630.

[bib65] Suni S. S. Brosi B. J., 2012 Population genetics of orchid bees in a fragmented tropical landscape. Conserv. Genet. 13: 323–332.

[bib66] Vogel S., 1966 Parfümsammelnde bienen als bestäuber von orchidaceen und Gloxinia. Österr. Bot. Z. 113: 302–361.

[bib67] Wcislo, W. T., and J. H. Cane, 1996 Floral resource utilization by solitary bees (Hymenoptera: Apoidea) and exploitation of their stored foods by natural enemies. Annu. Rev. Entomol. 41: 257–286. 10.1146/annurev.en.41.010196.001353 15012330

[bib68] Weber M. G. Mitko L. Eltz T. Ramírez S. R., 2016 Macroevolution of perfume signalling in orchid bees. Ecol. Lett. 19: 1314–1323. 27581155 10.1111/ele.12667

[bib69] Weinstock G. M. Robinson G. E. Gibbs R. A. Worley K. C. Evans J. D., 2006 Insights into social insects from the genome of the honeybee Apis mellifera. Nature 443: 931–949. 17073008 10.1038/nature05260 PMC2048586

[bib70] Whitten W. M. Young A. M. Stern D. L., 1993 Nonfloral sources of chemicals that attract male euglossine bees (Apidae: Euglossini). J. Chem. Ecol. 19: 3017–3027. 24248792 10.1007/BF00980599

[bib71] Woodard S. H. Fischman B. J. Venkat A. Hudson M. E. Varala K., 2011 Genes involved in convergent evolution of eusociality in bees. Proc. Natl. Acad. Sci. USA 108: 7472–7477. 21482769 10.1073/pnas.1103457108 PMC3088614

[bib72] Xu H. Luo X. Qian J. Pang X. Song J., 2012 FastUniq: a fast de novo duplicates removal tool for paired short reads. PLoS One 7: e52249. 23284954 10.1371/journal.pone.0052249 PMC3527383

[bib73] Zdobnov E. M. Tegenfeldt F. Kuznetsov D. Waterhouse R. M. Simão F. A., 2017 OrthoDB v9.1: cataloging evolutionary and functional annotations for animal, fungal, plant, archaeal, bacterial and viral orthologs. Nucleic Acids Res. 45: D744–D749. 27899580 10.1093/nar/gkw1119 PMC5210582

[bib74] Zimmermann Y. Schorkopf D. L. P. Moritz R. F. A. Pemberton R. W. Quezada-Euan J. J. G., 2011 Population genetic structure of orchid bees (Euglossini) in anthropogenically altered landscapes. Conserv. Genet. 12: 1183–1194.

